# A second ortho­rhom­bic polymorph of 2-(pyridin-4-ylmeth­oxy)phenol

**DOI:** 10.1107/S1600536812014067

**Published:** 2012-04-13

**Authors:** Guang-Tu Wang, Yong Zhang, Jin-Xin Yang, Ping Zou, Guang-Feng Hou

**Affiliations:** a College of Life Science, Sichuan Agriculture University, Ya’an 625014, People’s Republic of China; b Engineering Research Center of Pesticides of Heilongjiang University, Heilongjiang University, Harbin 150050, People’s Republic of China

## Abstract

The crystal structure of the title compound, C_12_H_11_NO_2_, represents a new ortho­rhom­bic polymorph II of the previously reported ortho­rhom­bic form I [Zhang *et al.* (2009 ▶) *Acta Cryst*. E**65**, o3160]. In polymorph II, the six-membered rings form a dihedral angle of 13.8 (1)° [71.6 (1)° in I], and O—H⋯N hydrogen bonds link mol­ecules into chains along [100], whereas the crystal structure of I features hydrogen-bonded centrosymmetric dimers.

## Related literature

For details of the synthesis, see: Gao *et al.* (2004 ▶). For the crystal structure of polymorph I, see: Zhang *et al.* (2009 ▶).
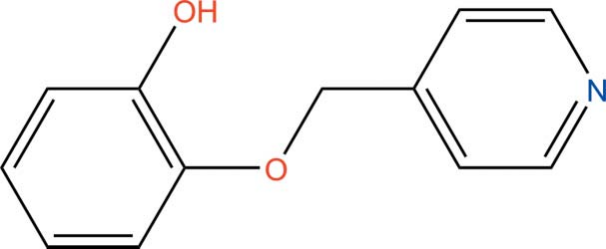


## Experimental

### 

#### Crystal data

C_12_H_11_NO_2_
*M* *_r_* = 201.22
Orthorhombic, 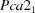

*a* = 23.398 (5) Å
*b* = 5.8343 (12) Å
*c* = 7.3934 (15) Å
*V* = 1009.3 (4) Å^3^
*Z* = 4
Mo *K*α radiation
μ = 0.09 mm^−1^
*T* = 293 K
0.50 × 0.37 × 0.11 mm

#### Data collection

Rigaku R-AXIS RAPID diffractometer
Absorption correction: multi-scan (*ABSCOR*; Higashi, 1995 ▶) *T*_min_ = 0.956, *T*_max_ = 0.990
8907 measured reflections
2285 independent reflections
1298 reflections with *I* > 2σ(*I*)
*R*_int_ = 0.066

#### Refinement

*R*[*F* ^2^ > 2σ(*F* ^2^)] = 0.043
*wR*(*F* ^2^) = 0.077
*S* = 1.01
2285 reflections
138 parameters
1 restraint
H-atom parameters constrained
Δρ_max_ = 0.15 e Å^−3^
Δρ_min_ = −0.14 e Å^−3^

### 

Data collection: *RAPID-AUTO* (Rigaku, 1998 ▶); cell refinement: *RAPID-AUTO*; data reduction: *CrystalClear* (Rigaku/MSC, 2002 ▶); program(s) used to solve structure: *SHELXS97* (Sheldrick, 2008 ▶); program(s) used to refine structure: *SHELXL97* (Sheldrick, 2008 ▶); molecular graphics: *SHELXTL* (Sheldrick, 2008 ▶); software used to prepare material for publication: *SHELXL97*.

## Supplementary Material

Crystal structure: contains datablock(s) I, global. DOI: 10.1107/S1600536812014067/cv5271sup1.cif

Structure factors: contains datablock(s) I. DOI: 10.1107/S1600536812014067/cv5271Isup2.hkl

Supplementary material file. DOI: 10.1107/S1600536812014067/cv5271Isup3.cml

Additional supplementary materials:  crystallographic information; 3D view; checkCIF report

## Figures and Tables

**Table 1:** Hydrogen-bond geometry (Å, °)

*D*—H⋯*A*	*D*—H	H⋯*A*	*D*⋯*A*	*D*—H⋯*A*
O1—H1⋯N1^i^	0.82	1.95	2.763 (2)	173

Symmetry code: (i) 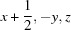.

**Fig. 1.**
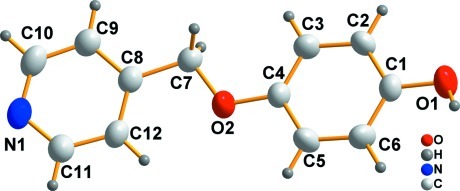
The molecular structure of the title compound, showing displacement ellipsoids at the 50% probability level for non-H atoms.

**Fig. 2.**
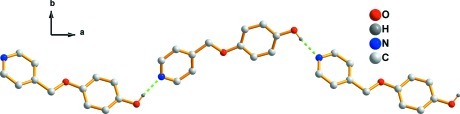
A partial packing view, showing hydrogen-bonded (dashed lines) chain structure along [100].

## supplementary crystallographic information

### Comment

The reported here crystal structure of the title compound, C_12_H_11_NO_2_, represents a new orthorhombic polymorph (II)of the previously reported orthorhombic form (I) (Zhang *et al.* 2009). It was crystalized from a methanol solution of the title compound and (*R*)-2-(4-(carboxymethoxy)phenoxy)propanoic acid mixture.

In polymorph II (Figure 1), two six-membered rings form a dihedral angle of 13.8 (1)° [71.6 (1)° in I], and intermolecular O—H···N hydrogen bonds link molecules into chains along [100] (Figure 2, Table 1), in spite of hydrogen-bonded centrosymmetric dimers in polymorph I.

### Experimental

The 2-(pyridin-4-ylmethoxy)phenol was synthesized by the reaction of *o*-benzenediol and 4-chloromethylpyridine hydrochloride under nitrogen atmosphere and alkaline condition (Gao *et al.*, 2004). Colourless block crystals were obtained by slow evaporation of a methanol solution (10 mL) containing title compound (0.402 g, 2 mmol) and (*R*)-2-(4-(carboxymethoxy)phenoxy)propanoic acid (0.48 g, 2 mmol) which only contained the molecules of title compound.

### Refinement

H atoms bound to C atoms and the H atoms of the hydroxyl groups were placed in calculated positions and treated as riding on their parent atoms, with C—H = 0.93 Å (aromatic), C—H = 0.97 Å (methylene), O—H = 0.82 Å and with U_iso_(H) = 1.2U_eq_(C), U_iso_(H) = 1.5U_eq_(O).

### Crystal data

**Table:**

C_12_H_11_NO_2_	*F*(000) = 424
*M**_r_* = 201.22	*D*_x_ = 1.324 Mg m^−^^3^
Orthorhombic, *P**c**a*2_1_	Mo *K*α radiation, λ = 0.71073 Å
Hall symbol: P 2c -2ac	Cell parameters from 5894 reflections
*a* = 23.398 (5) Å	θ = 3.3–27.5°
*b* = 5.8343 (12) Å	µ = 0.09 mm^−^^1^
*c* = 7.3934 (15) Å	*T* = 293 K
*V* = 1009.3 (4) Å^3^	Block, colourless
*Z* = 4	0.50 × 0.37 × 0.11 mm

### Data collection

**Table:**

Rigaku R-AXIS RAPID diffractometer	2285 independent reflections
Radiation source: fine-focus sealed tube	1298 reflections with *I* > 2σ(*I*)
Graphite monochromator	*R*_int_ = 0.066
ω scan	θ_max_ = 27.5°, θ_min_ = 3.3°
Absorption correction: multi-scan (*ABSCOR*; Higashi, 1995)	*h* = −30→30
*T*_min_ = 0.956, *T*_max_ = 0.990	*k* = −7→7
8907 measured reflections	*l* = −9→9

### Refinement

**Table:**

Refinement on *F*^2^	Secondary atom site location: difference Fourier map
Least-squares matrix: full	Hydrogen site location: inferred from neighbouring sites
*R*[*F*^2^ > 2σ(*F*^2^)] = 0.043	H-atom parameters constrained
*wR*(*F*^2^) = 0.077	*w* = 1/[σ^2^(*F*_o_^2^) + (0.0218*P*)^2^] where *P* = (*F*_o_^2^ + 2*F*_c_^2^)/3
*S* = 1.01	(Δ/σ)_max_ < 0.001
2285 reflections	Δρ_max_ = 0.15 e Å^−^^3^
138 parameters	Δρ_min_ = −0.14 e Å^−^^3^
1 restraint	Extinction correction: *SHELXL97* (Sheldrick, 2008), Fc^*^=kFc[1+0.001xFc^2^λ^3^/sin(2θ)]^-1/4^
Primary atom site location: structure-invariant direct methods	Extinction coefficient: 0.0028 (6)

### Special details

**Table:**

Geometry. All esds (except the esd in the dihedral angle between two l.s. planes) are estimated using the full covariance matrix. The cell esds are taken into account individually in the estimation of esds in distances, angles and torsion angles; correlations between esds in cell parameters are only used when they are defined by crystal symmetry. An approximate (isotropic) treatment of cell esds is used for estimating esds involving l.s. planes.
Refinement. Refinement of F^2^ against ALL reflections. The weighted R-factor wR and goodness of fit S are based on F^2^, conventional R-factors R are based on F, with F set to zero for negative F^2^. The threshold expression of F^2^ > 2sigma(F^2^) is used only for calculating R-factors(gt) etc. and is not relevant to the choice of reflections for refinement. R-factors based on F^2^ are statistically about twice as large as those based on F, and R- factors based on ALL data will be even larger.

### Fractional atomic coordinates and isotropic or equivalent isotropic displacement parameters (Å^2^)

**Table:**

	*x*	*y*	*z*	*U*_iso_*/*U*_eq_	
O1	0.68990 (6)	0.4700 (3)	0.0425 (3)	0.0567 (5)	
H1	0.7109	0.3578	0.0381	0.085*	
O2	0.46554 (6)	0.1873 (3)	−0.0201 (2)	0.0566 (5)	
N1	0.26917 (7)	−0.1195 (3)	0.0264 (3)	0.0488 (5)	
C2	0.59170 (9)	0.5453 (4)	0.0842 (3)	0.0442 (6)	
H2	0.6014	0.6865	0.1339	0.053*	
C8	0.36646 (8)	0.1609 (4)	0.0395 (3)	0.0390 (5)	
C7	0.41925 (8)	0.3104 (4)	0.0562 (3)	0.0429 (6)	
H7A	0.4137	0.4538	−0.0078	0.052*	
H7B	0.4269	0.3444	0.1824	0.052*	
C12	0.36737 (9)	−0.0462 (4)	−0.0507 (3)	0.0448 (6)	
H12	0.4003	−0.0958	−0.1091	0.054*	
C3	0.53418 (9)	0.4830 (4)	0.0713 (3)	0.0461 (6)	
H3	0.5057	0.5825	0.1107	0.055*	
C4	0.52013 (8)	0.2740 (4)	0.0000 (3)	0.0410 (6)	
C1	0.63411 (8)	0.4011 (4)	0.0247 (3)	0.0403 (5)	
C6	0.61922 (9)	0.1936 (4)	−0.0516 (4)	0.0518 (6)	
H6	0.6475	0.0958	−0.0948	0.062*	
C9	0.31568 (9)	0.2274 (4)	0.1199 (3)	0.0463 (6)	
H9	0.3132	0.3668	0.1804	0.056*	
C10	0.26894 (9)	0.0849 (4)	0.1093 (3)	0.0499 (7)	
H10	0.2351	0.1333	0.1629	0.060*	
C5	0.56238 (9)	0.1309 (4)	−0.0638 (4)	0.0541 (7)	
H5	0.5526	−0.0089	−0.1156	0.065*	
C11	0.31825 (8)	−0.1789 (4)	−0.0526 (3)	0.0485 (6)	
H11	0.3196	−0.3186	−0.1131	0.058*	

### Atomic displacement parameters (Å^2^)

**Table:**

	*U*^11^	*U*^22^	*U*^33^	*U*^12^	*U*^13^	*U*^23^
O1	0.0343 (8)	0.0535 (11)	0.0821 (13)	−0.0041 (7)	−0.0031 (10)	0.0010 (11)
O2	0.0315 (8)	0.0521 (10)	0.0861 (14)	−0.0023 (7)	0.0054 (9)	−0.0184 (11)
N1	0.0361 (10)	0.0534 (13)	0.0569 (12)	0.0003 (10)	0.0028 (10)	0.0084 (12)
C2	0.0414 (13)	0.0342 (13)	0.0569 (17)	−0.0032 (10)	−0.0020 (12)	−0.0076 (12)
C8	0.0350 (11)	0.0406 (13)	0.0416 (13)	0.0046 (10)	−0.0013 (11)	0.0052 (12)
C7	0.0378 (12)	0.0438 (13)	0.0472 (15)	0.0026 (10)	0.0045 (12)	−0.0016 (14)
C12	0.0333 (12)	0.0500 (15)	0.0510 (14)	0.0030 (11)	0.0049 (12)	0.0009 (14)
C3	0.0413 (13)	0.0392 (14)	0.0577 (16)	0.0057 (11)	0.0009 (13)	−0.0070 (13)
C4	0.0313 (11)	0.0430 (13)	0.0487 (14)	0.0001 (10)	0.0011 (10)	−0.0034 (12)
C1	0.0329 (11)	0.0421 (13)	0.0460 (14)	−0.0004 (10)	0.0000 (12)	0.0045 (13)
C6	0.0375 (12)	0.0489 (15)	0.0690 (17)	0.0089 (11)	0.0008 (14)	−0.0165 (15)
C9	0.0425 (13)	0.0437 (15)	0.0528 (15)	0.0036 (12)	0.0052 (11)	0.0025 (13)
C10	0.0367 (13)	0.0554 (17)	0.0577 (16)	0.0096 (12)	0.0054 (12)	0.0097 (15)
C5	0.0424 (13)	0.0429 (14)	0.0771 (18)	−0.0008 (11)	−0.0026 (14)	−0.0206 (16)
C11	0.0432 (12)	0.0516 (16)	0.0506 (14)	−0.0007 (12)	0.0004 (12)	−0.0006 (14)

### Geometric parameters (Å, º)

**Table:**

O1—C1	1.372 (2)	C12—C11	1.386 (3)
O1—H1	0.8200	C12—H12	0.9300
O2—C4	1.382 (2)	C3—C4	1.368 (3)
O2—C7	1.417 (2)	C3—H3	0.9300
N1—C11	1.334 (3)	C4—C5	1.377 (3)
N1—C10	1.340 (3)	C1—C6	1.380 (3)
C2—C1	1.373 (3)	C6—C5	1.382 (3)
C2—C3	1.397 (3)	C6—H6	0.9300
C2—H2	0.9300	C9—C10	1.376 (3)
C8—C12	1.381 (3)	C9—H9	0.9300
C8—C9	1.384 (3)	C10—H10	0.9300
C8—C7	1.517 (3)	C5—H5	0.9300
C7—H7A	0.9700	C11—H11	0.9300
C7—H7B	0.9700		
			
C1—O1—H1	109.5	C3—C4—C5	119.98 (18)
C4—O2—C7	118.54 (17)	C3—C4—O2	126.16 (19)
C11—N1—C10	115.8 (2)	C5—C4—O2	113.8 (2)
C1—C2—C3	121.0 (2)	O1—C1—C2	118.5 (2)
C1—C2—H2	119.5	O1—C1—C6	122.45 (19)
C3—C2—H2	119.5	C2—C1—C6	119.08 (19)
C12—C8—C9	117.8 (2)	C1—C6—C5	120.1 (2)
C12—C8—C7	122.01 (19)	C1—C6—H6	119.9
C9—C8—C7	120.2 (2)	C5—C6—H6	119.9
O2—C7—C8	107.36 (18)	C10—C9—C8	119.2 (2)
O2—C7—H7A	110.2	C10—C9—H9	120.4
C8—C7—H7A	110.2	C8—C9—H9	120.4
O2—C7—H7B	110.2	N1—C10—C9	124.1 (2)
C8—C7—H7B	110.2	N1—C10—H10	118.0
H7A—C7—H7B	108.5	C9—C10—H10	118.0
C8—C12—C11	118.8 (2)	C4—C5—C6	120.5 (2)
C8—C12—H12	120.6	C4—C5—H5	119.7
C11—C12—H12	120.6	C6—C5—H5	119.7
C4—C3—C2	119.3 (2)	N1—C11—C12	124.4 (2)
C4—C3—H3	120.3	N1—C11—H11	117.8
C2—C3—H3	120.3	C12—C11—H11	117.8

### Hydrogen-bond geometry (Å, º)

**Table:**

*D*—H···*A*	*D*—H	H···*A*	*D*···*A*	*D*—H···*A*
O1—H1···N1^i^	0.82	1.95	2.763 (2)	173

Symmetry code: (i) *x*+1/2, −*y*, *z*.
