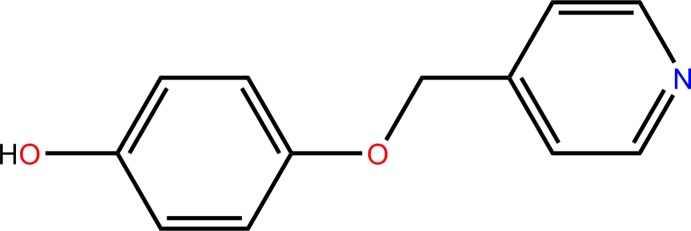# A second ortho­rhom­bic polymorph of 2-(pyridin-4-ylmeth­oxy)phenol. Corrigendum

**DOI:** 10.1107/S1600536812027225

**Published:** 2012-06-30

**Authors:** Guang-Tu Wang, Yong Zhang, Jin-Xin Yang, Ping Zou, Guang-Feng Hou

**Affiliations:** a College of Life Science, Sichuan Agriculture University, Ya’an 625014, People’s Republic of China; b Engineering Research Center of Pesticides of Heilongjiang University, Heilongjiang University, Harbin 150050, People’s Republic of China

## Abstract

The title and Scheme in the paper by Wang *et al.* [*Acta Cryst.* (2012), E**68**, o1366] are corrected.

## Abstract 2

Corrigendum to *Acta Cryst.* (2012), E**68**, o1366.

In the paper by Wang *et al.* (2012) ▶, the chemical name is incorrect in the title and, as a result, the statement in the title is also incorrect. The correct title should be ‘4-(Pyridin-4-ylmeth­oxy)phenol’. The correct Scheme is shown below.